# 3-Hy­droxy­pyridinium-2-carboxylate

**DOI:** 10.1107/S1600536811027462

**Published:** 2011-07-16

**Authors:** Richard Betz, Thomas Gerber

**Affiliations:** a Nelson Mandela Metropolitan University, Summerstrand Campus, Department of Chemistry, University Way, Summerstrand, PO Box 77000, Port Elizabeth, 6031, South Africa

## Abstract

Comparable to many amino acids, the title compound, C_6_H_5_NO_3_, is a substitution product of picolinic acid. The mol­ecule shows approximate non-crystallographic *C_s_* symmetry. Like many amino acids, the mol­ecule is present in its zwitterionic state. Intra- as well as inter­molecular hydrogen bonds are observed, the latter connecting the mol­ecules into zigzag chains along the crystallographic *b* axis. An inter­molecular C—C distance of only 3.368 (2) Å exclusively involving carbon atoms of aromatic rings (centroid–centroid separation = 3.803 Å) is indicative of π–π inter­actions connecting the mol­ecules into stacks along the crystallographic *a* axis.

## Related literature

For the use of chelate ligands as opposed to monodentate ligands, see: Gade (1998 ▶). For the crystal structures of two mercury coordination compounds applying the title compound as a mono-, as well as a bidentate, ligand, see: Popović *et al.* (2007 ▶). For graph-set analysis of hydrogen bonds, see: Etter *et al.* (1990 ▶); Bernstein *et al.* (1995 ▶).
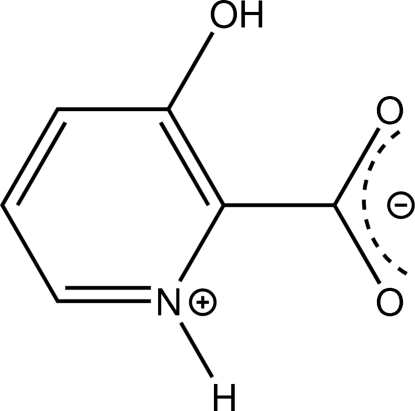


## Experimental

### 

#### Crystal data

C_6_H_5_NO_3_
*M* *_r_* = 139.11
Monoclinic, 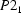

*a* = 3.8034 (1) Å
*b* = 6.8144 (2) Å
*c* = 11.1807 (4) Å
β = 95.102 (1)°
*V* = 288.63 (2) Å^3^
*Z* = 2
Mo *K*α radiation
μ = 0.13 mm^−1^
*T* = 200 K
0.56 × 0.50 × 0.22 mm

#### Data collection

Bruker APEXII CCD diffractometer
2659 measured reflections
768 independent reflections
758 reflections with *I* > 2σ(*I*)
*R*_int_ = 0.029

#### Refinement

*R*[*F* ^2^ > 2σ(*F* ^2^)] = 0.030
*wR*(*F* ^2^) = 0.083
*S* = 1.07
768 reflections
96 parameters
1 restraint
H atoms treated by a mixture of independent and constrained refinement
Δρ_max_ = 0.26 e Å^−3^
Δρ_min_ = −0.15 e Å^−3^

### 

Data collection: *APEX2* (Bruker, 2010 ▶); cell refinement: *SAINT* (Bruker, 2010 ▶); data reduction: *SAINT*; program(s) used to solve structure: *SHELXS97* (Sheldrick, 2008 ▶); program(s) used to refine structure: *SHELXL97* (Sheldrick, 2008 ▶); molecular graphics: *ORTEPIII* (Farrugia, 1997 ▶) and *Mercury* (Macrae *et al.*, 2008 ▶); software used to prepare material for publication: *SHELXL97* and *PLATON* (Spek, 2009 ▶).

## Supplementary Material

Crystal structure: contains datablock(s) I, global. DOI: 10.1107/S1600536811027462/ez2254sup1.cif

Supplementary material file. DOI: 10.1107/S1600536811027462/ez2254Isup2.cdx

Structure factors: contains datablock(s) I. DOI: 10.1107/S1600536811027462/ez2254Isup3.hkl

Supplementary material file. DOI: 10.1107/S1600536811027462/ez2254Isup4.cml

Additional supplementary materials:  crystallographic information; 3D view; checkCIF report

## Figures and Tables

**Table 1:** Hydrogen-bond geometry (Å, °)

*D*—H⋯*A*	*D*—H	H⋯*A*	*D*⋯*A*	*D*—H⋯*A*
O3—H3⋯O1	0.84	1.75	2.4997 (17)	148
N1—H71⋯O2^i^	1.01 (2)	1.80 (3)	2.6767 (17)	143 (3)

Symmetry code: (i) 
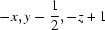
.

**Fig. 1.**
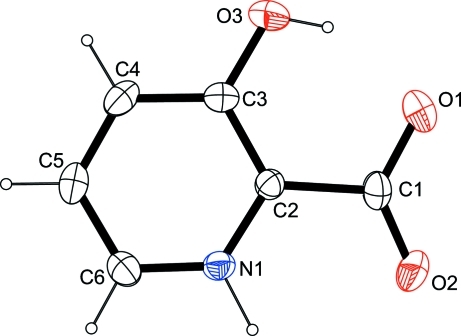
The molecular structure of the title compound, with atom labels and anisotropic displacement ellipsoids (drawn at 50% probability level).

**Fig. 2.**
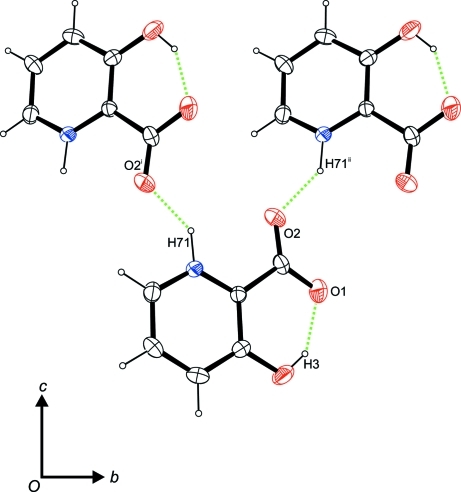
Hydrogen bonds, indicated by green dashed lines, viewed along [-1 0 0]. Symmetry operators: i -x, y - 1/2, -z + 1; ii -x, y + 1/2, -z + 1.

**Fig. 3.**
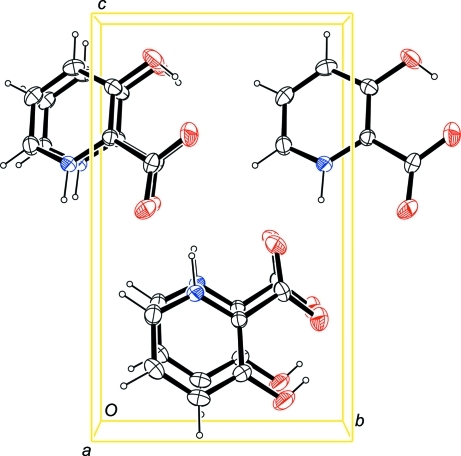
Molecular packing of the title compound, viewed along [-1 0 0] (anisotropic displacement ellipsoids drawn at 50% probability level).

## supplementary crystallographic information

### Comment

Chelate ligands have found widespread use in coordination chemistry due to the enhanced thermodynamic stability of resultant coordination compounds in relation to coordination compounds exclusively applying comparable monodentate ligands (Gade, 1998). Combining two different donor atoms in different states of hybridization might be useful to accomodate a large variety of metal centers of variable Lewis acidity. In this aspect, 3-hyxdroxypicolinic acid seemed of interest due to its possible use as a strictly neutral or, depending on the pH value, as an anionic or cationic ligand. In addition, due to the arrangement of its functional groups, it may act as mono- or bidentate ligand offering the possibility to create five- as well as six-membered chelate rings. To enable comparative studies in terms of bond lengths and angles in envisioned coordination compounds, we determined the molecular and crystal structure of the title compound. Among a few others, the crystal structures of two mercury coordination compounds in which 3-hydroxypicolinic acid acts as mono- or bidentate ligand exist in the literature (Popović *et al.*, 2007).

The molecule (Fig. 1) is present in its zwitterionic tautomeric form and thus resembles natural amino acids. Intracyclic angles span a range of 118.48 (14)–123.88 (12) ° with the biggest angle found on the protonated nitrogen atom. Nearly all atoms of the molecule are in plane. The least-squares planes defined by the aromatic moiety on the one hand and the atoms of the carboxylic acid group on the other hand enclose an angle of only 2.8 (3) °.

Apart from an intramolecular hydrogen bond obvious between the hydroxyl group and the carboxylic acid group, intermolecular hydrogen bonds are observed (Fig. 2). These stem from the protonated nitrogen atom and have one of the carboxylic acid group's oxygen atoms as acceptor. In terms of graph-set analysis (Etter *et al.*, 1990; Bernstein *et al.*, 1995), the descriptor for this hydrogen bonding system on the unitary level is *DC*^1^_1_(5). In total, the molecules are connected to waved zigzag chains along the crystallographic *b* axis. The presence of π···π interactions becomes manifest upon the presence of an intermolecular C–C distance of only 3.368 (2) Å. This interaction exclusively involves intracyclic carbon atoms and gives rise to stacks of molecules along the crystallographic *a* axis.

The packing of the compound is shown in Fig. 3.

### Experimental

The compound was obtained commercially (Fluka). Crystals suitable for the diffraction study were obtained upon recrystallization of the compound from hot water.

### Refinement

Carbon-bound H atoms were placed in calculated positions (C—H 0.95 Å) and were included in the refinement in the riding model approximation, with *U*(H) set to 1.2*U*_eq_(C). The hydrogen atom of the hydroxyl group was allowed to rotate with a fixed angle around the O–C bond to best fit the experimental electron density (HFIX 147 in the *SHELX* program suite (Sheldrick, 2008). The hydrogen atom of the protonated nitrogen atom was located on a difference Fourier map and refined freely.

### Crystal data

**Table:**

C_6_H_5_NO_3_	*F*(000) = 144
*M**_r_* = 139.11	*D*_x_ = 1.601 Mg m^−^^3^
Monoclinic, *P*2_1_	Mo *K*α radiation, λ = 0.71073 Å
Hall symbol: P 2yb	Cell parameters from 2465 reflections
*a* = 3.8034 (1) Å	θ = 3.0–28.3°
*b* = 6.8144 (2) Å	µ = 0.13 mm^−^^1^
*c* = 11.1807 (4) Å	*T* = 200 K
β = 95.102 (1)°	Block, colourless
*V* = 288.63 (2) Å^3^	0.56 × 0.50 × 0.22 mm
*Z* = 2	

### Data collection

**Table:**

Bruker APEXII CCD diffractometer	758 reflections with *I* > 2σ(*I*)
Radiation source: fine-focus sealed tube	*R*_int_ = 0.029
graphite	θ_max_ = 28.3°, θ_min_ = 1.8°
φ and ω scans	*h* = −5→3
2659 measured reflections	*k* = −9→9
768 independent reflections	*l* = −14→14

### Refinement

**Table:**

Refinement on *F*^2^	Primary atom site location: structure-invariant direct methods
Least-squares matrix: full	Secondary atom site location: difference Fourier map
*R*[*F*^2^ > 2σ(*F*^2^)] = 0.030	Hydrogen site location: inferred from neighbouring sites
*wR*(*F*^2^) = 0.083	H atoms treated by a mixture of independent and constrained refinement
*S* = 1.07	*w* = 1/[σ^2^(*F*_o_^2^) + (0.0544*P*)^2^ + 0.0435*P*] where *P* = (*F*_o_^2^ + 2*F*_c_^2^)/3
768 reflections	(Δ/σ)_max_ < 0.001
96 parameters	Δρ_max_ = 0.26 e Å^−^^3^
1 restraint	Δρ_min_ = −0.15 e Å^−^^3^

### Special details

**Table:**

Refinement. Due to the absence of a strong anomalous scatterer, the Flack parameter is meaningless. Thus, Friedel opposites (588 pairs) have been merged and the item was removed from the CIF.

### Fractional atomic coordinates and isotropic or equivalent isotropic displacement parameters (Å^2^)

**Table:**

	*x*	*y*	*z*	*U*_iso_*/*U*_eq_	
O1	−0.0877 (3)	0.8750 (2)	0.28327 (12)	0.0355 (3)	
O2	−0.0543 (4)	0.7074 (2)	0.45701 (11)	0.0357 (3)	
O3	0.1674 (4)	0.7340 (2)	0.10355 (11)	0.0350 (3)	
H3	0.0796	0.8189	0.1465	0.053*	
N1	0.2583 (3)	0.4001 (2)	0.35230 (10)	0.0207 (3)	
H71	0.188 (7)	0.386 (5)	0.437 (2)	0.051 (7)*	
C1	−0.0040 (4)	0.7302 (2)	0.35008 (14)	0.0242 (3)	
C2	0.1756 (4)	0.5650 (2)	0.28933 (12)	0.0191 (3)	
C3	0.2518 (4)	0.5751 (2)	0.16925 (12)	0.0226 (3)	
C4	0.4148 (4)	0.4133 (3)	0.11965 (12)	0.0270 (4)	
H4	0.4699	0.4169	0.0385	0.032*	
C5	0.4942 (4)	0.2504 (3)	0.18837 (14)	0.0274 (3)	
H5	0.6056	0.1411	0.1549	0.033*	
C6	0.4120 (4)	0.2445 (3)	0.30745 (14)	0.0254 (3)	
H6	0.4648	0.1315	0.3556	0.030*	

### Atomic displacement parameters (Å^2^)

**Table:**

	*U*^11^	*U*^22^	*U*^33^	*U*^12^	*U*^13^	*U*^23^
O1	0.0439 (7)	0.0223 (6)	0.0408 (7)	0.0096 (6)	0.0061 (5)	0.0009 (5)
O2	0.0495 (7)	0.0310 (7)	0.0287 (5)	0.0057 (6)	0.0149 (5)	−0.0076 (5)
O3	0.0464 (7)	0.0322 (7)	0.0274 (6)	0.0076 (6)	0.0081 (5)	0.0125 (6)
N1	0.0247 (6)	0.0195 (6)	0.0182 (5)	−0.0026 (5)	0.0037 (4)	0.0009 (5)
C1	0.0254 (7)	0.0189 (7)	0.0288 (7)	0.0011 (6)	0.0046 (5)	−0.0054 (6)
C2	0.0206 (6)	0.0175 (6)	0.0196 (6)	−0.0010 (5)	0.0034 (4)	−0.0007 (6)
C3	0.0240 (6)	0.0236 (8)	0.0202 (6)	−0.0008 (6)	0.0021 (5)	0.0034 (6)
C4	0.0272 (7)	0.0352 (9)	0.0194 (6)	0.0013 (7)	0.0061 (5)	−0.0038 (7)
C5	0.0278 (7)	0.0261 (8)	0.0285 (7)	0.0035 (7)	0.0045 (5)	−0.0087 (7)
C6	0.0282 (7)	0.0196 (7)	0.0281 (7)	0.0007 (6)	0.0012 (5)	0.0014 (6)

### Geometric parameters (Å, °)

**Table:**

O1—C1	1.261 (2)	C2—C3	1.4002 (17)
O2—C1	1.237 (2)	C3—C4	1.403 (2)
O3—C3	1.332 (2)	C4—C5	1.369 (3)
O3—H3	0.8400	C4—H4	0.9500
N1—C6	1.330 (2)	C5—C6	1.395 (2)
N1—C2	1.348 (2)	C5—H5	0.9500
N1—H71	1.01 (2)	C6—H6	0.9500
C1—C2	1.510 (2)		
			
?···?	?		
			
C3—O3—H3	109.5	O3—C3—C4	120.95 (13)
C6—N1—C2	123.88 (12)	C2—C3—C4	118.48 (14)
C6—N1—H71	115.9 (19)	C5—C4—C3	119.92 (12)
C2—N1—H71	120.1 (19)	C5—C4—H4	120.0
O2—C1—O1	128.19 (15)	C3—C4—H4	120.0
O2—C1—C2	117.13 (15)	C4—C5—C6	120.15 (15)
O1—C1—C2	114.68 (13)	C4—C5—H5	119.9
N1—C2—C3	118.89 (12)	C6—C5—H5	119.9
N1—C2—C1	118.73 (12)	N1—C6—C5	118.68 (15)
C3—C2—C1	122.36 (13)	N1—C6—H6	120.7
O3—C3—C2	120.57 (13)	C5—C6—H6	120.7
			
C6—N1—C2—C3	0.5 (2)	N1—C2—C3—C4	−0.6 (2)
C6—N1—C2—C1	179.01 (13)	C1—C2—C3—C4	−179.10 (14)
O2—C1—C2—N1	2.6 (2)	O3—C3—C4—C5	−179.02 (14)
O1—C1—C2—N1	−176.69 (13)	C2—C3—C4—C5	0.2 (2)
O2—C1—C2—C3	−178.94 (14)	C3—C4—C5—C6	0.3 (2)
O1—C1—C2—C3	1.8 (2)	C2—N1—C6—C5	0.1 (2)
N1—C2—C3—O3	178.64 (14)	C4—C5—C6—N1	−0.5 (2)
C1—C2—C3—O3	0.2 (2)		

### Hydrogen-bond geometry (Å, °)

**Table:**

*D*—H···*A*	*D*—H	H···*A*	*D*···*A*	*D*—H···*A*
O3—H3···O1	0.84	1.75	2.4997 (17)	148.
N1—H71···O2^i^	1.01 (2)	1.80 (3)	2.6767 (17)	143 (3)

Symmetry codes: (i) −*x*, *y*−1/2, −*z*+1.
